# The Multiple Roles of the Cdc14 Phosphatase in Cell Cycle Control

**DOI:** 10.3390/ijms21030709

**Published:** 2020-01-21

**Authors:** Javier Manzano-López, Fernando Monje-Casas

**Affiliations:** Centro Andaluz de Biología Molecular y Medicina Regenerativa (CABIMER), Spanish National Research Council (CSIC)—University of Seville—University Pablo de Olavide, 41092 Sevilla, Spain; javier.manzano@cabimer.es

**Keywords:** Cdc14, phosphatase, mitotic exit, genome stability, nucleolus, autophagy, cytokinesis

## Abstract

The Cdc14 phosphatase is a key regulator of mitosis in the budding yeast *Saccharomyces cerevisiae*. Cdc14 was initially described as playing an essential role in the control of cell cycle progression by promoting mitotic exit on the basis of its capacity to counteract the activity of the cyclin-dependent kinase Cdc28/Cdk1. A compiling body of evidence, however, has later demonstrated that this phosphatase plays other multiple roles in the regulation of mitosis at different cell cycle stages. Here, we summarize our current knowledge about the pivotal role of Cdc14 in cell cycle control, with a special focus in the most recently uncovered functions of the phosphatase.

## 1. Introduction

The cell cycle comprises a series of processes that ensure the duplication of the genome and the cellular content, as well as their safe partitioning between the two newly generated daughter cells. Among other mechanisms, the coordination between these events is safeguarded by an accurate balance between kinases and phosphatases that regulate the phosphorylation status of the proteins that control the progression through mitosis. In the budding yeast *Saccharomyces cerevisiae*, the Cdc14 phosphatase, originally described in the pioneer screening carried out by Hartwell et al. [1], is a master regulator of many essential cell cycle events.

The phosphatases from the Cdc14 family show an extraordinary evolutionary conservation. These enzymes are probably the best-characterized members of the dual-specificity phosphatases group, which comprises proteins with the ability to dephosphorylate both phospho-serine (pSer)/phospho-threonine (pThr) and phospho-tyrosine (pTyr) residues [2,3]. The N-terminal region of Cdc14 phosphatases is highly conserved and includes two domains that are essential for their enzymatic activity: an A domain, which contributes to substrate specificity, and a catalytic B domain [4]. The C-terminal region is more variable, although it carries a nuclear export sequence (NES) that is representative of this part of the protein. These phosphatases also have a nuclear localization sequence (NLS) that is located in their N-terminal region in higher eukaryotes, but is found in the C-end of the budding yeast homolog [2,5]. The NES and NLS domains are crucial for the regulation of Cdc14 phosphatases, as the activity of these proteins is controlled by changes in their subcellular localization. As such, in *S. cerevisiae*, Cdc14 is sequestered in the nucleolus during most of the cell cycle, from where it is released at the onset of anaphase [6]. Traditionally, once that Cdc14 is released, the main role assigned to this protein in the control of the cell cycle in budding yeast is to counteract the activity of cyclin-dependent kinase (Cdk) complexes, the main drivers of entry and progression through the cell cycle [6,7]. Accordingly, the absence of this phosphatase blocks mitotic exit and halts the cell cycle in anaphase with cells displaying elevated levels of Cdc28/Cdk1 activity, the only Cdk present in *S. cerevisiae* [8]. However, Cdc14 has many other functions in cell cycle control in this organism, which makes this phosphatase a protein of a pivotal importance as a mitotic regulator. In fact, and in contrast to what described for budding yeast, Clp1/Flp1, the Cdc14 homologue in the fission yeast *Schizzosaccharomyces pombe*, is not essential for accomplishing mitotic exit and it is instead required for the proper execution of cytokinesis [9,10]. As for Cdc14, regulation of Clp1/Flp1 is also greatly dependent on the control of its cellular localization. However, although Clp1/Flp1 is maintained sequestered in the nucleolus, it differs in the timing of its release from this cellular compartment, as Clp1/Flp1 is retained during S phase but liberated soon as cells enter mitosis [9,10].

Metazoan homologues of Cdc14 also change their localization during the cell cycle. Among them, the best characterized are the human hCdc14A and hCdc14B phosphatases, which have been implicated in the regulation of cell cycle progression, centriole duplication, mitotic exit, cytokinesis, actin organization, and cell migration and adhesion [11,12,13,14,15,16,17,18,19]. hCdc14A and hCdc14B display different localization. In this way, while hCdc14A locates to centrosomes in interphase, hCdc14B is predominantly a nucleolar protein that migrates in mitosis to microtubules, the midbody, and the spindle midzone [18,19]. Cdc14 function seems to also be regulated in a cell cycle-dependent manner in humans, as hCdc14A phosphorylation by Cdk1 modulates the binding pattern of the phosphatase to its interactors [20]. Remarkably, human Cdc14 homologue expression is able to rescue the phenotype associated to Cdc14 depletion in both fission and budding yeast [21,22]. Interestingly, hCdc14A interacts in vivo and in vitro with the tumor suppressor p53 in order to promote its dephosphorylation at Ser315, as well as with the Cdc2/cyclin B complex [23,24]. Moreover, hCdc14A is differentially expressed in multiple human cancer cell lines and, remarkably, the phosphatase levels in these cell lines inversely correlate to those of p53 [24]. These results suggest that deregulation of the Cdc14 phosphatase homologues in humans could contribute to tumorigenesis. Because, as previously mentioned, Cdc14 phosphatases are also involved in actin organization, cell migration and adhesion [16,17], ciliogenesis [25,26], centrosome duplication, and chromosome segregation [11,19]—all processes associated with tumor progression and metastasis—future research increasing our knowledge about the structure, function, and regulation of this conserved family of phosphatases will be of great value to better understand the mechanisms leading to tumorigenesis and, hence, to develop new therapies against this disease.

**Figure 1:**
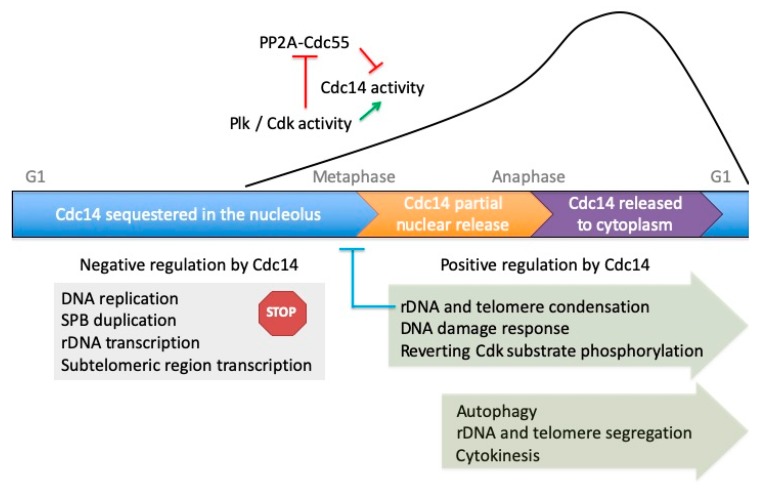
Cdc14 phosphatase is sequestered into the nucleolus through binding to Net1/Cfi1 [6]. While inactive in this cellular compartment, Cdc14 cannot initiate DNA and spindle pole body (SPB) reduplication cycles [27,28,43,44], and the phosphorylated state of the RNA polymerases that preserve the transcriptional activity of sub-telomeric and ribosomal DNA (rDNA) regions can be maintained [34]. At the metaphase-to-anaphase transition, FEAR together with Plk and Cdk downregulate PP2A-Cdc55 (inhibitory red line) and trigger Net1/Cfi1 phosphorylation to promote the first releasing wave of Cdc14 into the nucleus (green arrow) [45,46]. Once at this location, Cdc14 activity (black line) progressively downregulates cyclin-dependent kinase (Cdk) phosphorylation, thereby promoting rDNA and telomere condensation through RNA polymerase I and II inhibition to facilitate the segregation of these chromosomal regions [33,34,47,48] and also priming the activation of the mitotic exit network (MEN) pathway [49,50]. Nucleolar hyper-condensation blocks efficient Cdc14 release (inhibitory blue line) [32]. Cdc14 further participates in the DNA damage response by anchoring DNA lesions to the SPBs, in this way facilitating their repair [51]. In anaphase, Cdc55 phosphorylation by Cdk1-Clb2 stimulates full Net1/Cfi1 and Cdc14 disassociation and, subsequently, completes Cdc14 activation (inhibitory red line) [52]. The final release of Cdc14 to the cytoplasm globally reverses Cdk activity in coordination with PPA2-Cdc55 and PPA2-Rts1 phosphatases, supporting exit from mitosis [53]. Excitingly, Cdc14 also works as a central hub that coordinates mitotic progression with signaling pathways such as high-osmolarity glycerol (HOG) or target of rapamycin complex 1 (TORC1) [54,55,56]. Notably, however, Cdc14 functions are not restricted to cell cycle regulation, as the phosphatase can also induce autophagy under starvation stress [42].

Therefore, the research accumulated over recent years demonstrates that besides mitotic progression, and more specifically the regulation of mitotic exit, many other fundamental processes are also under the control of proteins from the Cdc14 family, from yeast to metazoans. In this review, we aim to summarize and disclose all these novel aspects of Cdc14 regulation and functions (Figure 1), which include from centrosome duplication [12,27,28] to DNA repair [29,30,31]; chromosome compaction, segregation, or transcription [32,33,34,35,36]; cytokinesis [37,38]; morphogenesis [39,40,41]; and autophagy [42], among others.

## 2. Cdc14 and the Regulation of the Cell Cycle

The role of Cdc14 in Cdk inactivation and cell cycle control has been extensively studied in the budding yeast *Saccharomyces cerevisiae*. In this organism, Cdc14 is sequestered in the nucleolus from G1 to metaphase through its binding to Net1/Cfi1 [6], to then be released from this cellular compartment in two sequential waves: first, the phosphatase moves from the nucleolus to the nucleoplasm in early anaphase and, subsequently, it is delivered from the nucleus into the cytoplasm in late anaphase [8] (Figure 1). The initial nucleolar liberation of Cdc14 during the early anaphase stages is supported by the FEAR (Cdc-Fourteen early anaphase release) pathway, which promotes Net1/Cfi1 phosphorylation by both the polo-like kinase Cdc5 and Cdc28/Cdk1 [45,57,58,59]. On the other hand, the final release of the phosphatase is dependent on the MEN (mitotic exit network) pathway, a signaling cascade that is initiated by the Tem1 GTPase and that finally promotes the phosphorylation of the NLS in Cdc14 by the Dbf2-Mob1 kinase, thus favoring its cytoplasmic liberation in the late stages of anaphase [5,8,60,61]. Cdc5 plays a dual role in Cdc14 release as part of both the FEAR and the MEN. Interestingly, recent insights into this process suggest that Cdc5 is sequentially phosphorylated by Clb2-Cdk1 in order to promote the successive waves of Cdc14 release [62]. Cdc14 sequential liberation is of paramount importance during the metaphase-to-anaphase transition for spindle stabilization and elongation, activation of the MEN, mitotic exit, and cytokinesis [45,49,63,64,65,66] (Figure 1).

At the end of mitosis, Cdc14 is re-sequestered in the nucleolus by a not yet completely characterized mechanism that is mediated by Cdc5 degradation [67]. The decay of Cdk activity is also essential for Cdc14 inactivation at the end of mitosis, as re-sequestration of the phosphatase is bypassed when Cdk activity is maintained constant. Under these conditions, Cdc5 is expressed again after its degradation and, subsequently, it can trigger a new wave of Cdc14 release that eventually determines a novel round of polo-like kinase degradation. This interplay between Cdk, Cdc5, and Cdc14 unveils an autonomous oscillator cycle that controls the nucleolar release of the Cdc14 phosphatase [57,68].

Premature release of Cdc14 is prevented by two different mechanisms. First, PP2A-Cdc55 phosphatase counteracts Cdk activity until metaphase, which precludes its action on Cdc14 and Net1/Cfi1 [46,69]. Second, the Kin4 kinase blocks exit from mitosis until the spindle is correctly positioned along the mother–daughter polarity axis by preventing the Cdc5-dependent activation of the MEN [70,71]. At anaphase onset, PP2A-Cdc55 is downregulated in a separase-dependent manner in cooperation with Zds1/Zds2 [64]. Finally, during anaphase, Clb2-Cdk1 promotes Cdc55 subunit phosphorylation to inhibit the phosphatase activity of PP2A and, once that the spindle is properly positioned, cells initiate mitotic exit signaling [52,70,71].

Cdc14 shows a strong affinity for pSer-Pro-X-Lys>Arg motifs in vitro. This peptide is a phosphorylation target for Cdk [29,72]. The affinity of Cdc14 for this motif has also been recently validated by in vivo experiments [53,73]. Interestingly, recent findings suggest that Cdc14 acts as a dimer and that dimerization is essential to reach an efficient phosphatase activity [74,75]. Three Cdc14 residues are critical for dimer formation: P123, which is located at the periphery of the interface and is relevant for hydrophobic interactions, and Y416 and Y330, which promote monomer contacts. Accordingly, P123E and Y146K/Y330K mutations severely compromise cell growth [74]. Additionally, it has been shown that Cdc14 recognizes Pro-X-Leu motifs as docking sites in its substrates to favor their interaction and thus its phosphatase activity [75]. Remarkably, the hydrophobic pocket of Cdc14 that interacts with the Pro-X-Leu motif is conserved in Cdc14 human orthologues [75]. Appealingly, the Net1-Cdc14 interaction could be also mediated by the presence of a docking domain and, subsequently, Net1/Cfi1 could act as a pseudo-substrate inhibitor [75]. In budding yeast, numerous Cdc14 substrates have been identified, including cell-cycle regulators such as the Cdk inhibitor Sic1 and its transcription factor Swi5, the MEN components Bfa1 and Cdc15, or the Swe1 kinase [7,49,50,76]. In agreement with a role of Cdc14 in the stabilization of the spindle midzone and the elongation of the spindle in anaphase, microtubule-associated proteins such as Ase1, Ask1, Fin1, or Sli15 are also substrates of this phosphatase [77,78,79,80]. Similarly, kinetochore proteins are preferential substrates for Cdc14 and are subsequently also under regulation of this phosphatase [54,81,82]. Interestingly, the Hsp90 chaperone is dephosphorylated by Cdc14 in yeast, with this being necessary to promote the dissociation of Hsp90 from the Mps1 kinase, which is a requisite for mitotic exit [83]. Likewise, another relevant substrate whose Cdc14-dependent dephosphorylation favors mitotic exit is the nuclear rim protein Nur1 [84]. Finally, Cdc14 also dephosphorylates substrates that are important for septin ring splitting and constriction of the actomyosin ring during cytokinesis [85]. In fact, and interestingly, despite reversal of Cdk phosphorylation and inhibition of its kinase activity being the main role described for Cdc14 in budding yeast, the function of the phosphatase in the regulation of cytokinesis seems however to be the most evolutionary conserved among the Cdc14 family [73].

As previously indicated, and in agreement with its essential role in Cdk inhibition, *S. cerevisiae* cells carrying conditional null alleles of *cdc14* arrest in anaphase with high levels of Cdk activity [7]. Interestingly, the imbalance between the differential affinities that Cdk and Cdc14 display towards their substrates also contributes to the sequential completion of the different steps that lead to mitotic exit. In this sense, the equilibrium between kinase and phosphatase activities could also be determined by the presence of a Pro-X-Leu docking site, which might contribute to establish the dephosphorylating timing [75]. Accordingly, proteins that participate in chromosome segregation and anaphase spindle elongation are dephosphorylated before substrates involved in spindle disassembly, replication origin re-licensing, and return to the G1 phase [86]. Despite this, and remarkably, a recent report using an auxin-based Cdc14 degron showed that mitotic exit could take place after conditional depletion of the phosphatase [73]. This unexpected result could however be explained according to a different study that demonstrates that residual levels of Cdc14 can be compensated by the activity of other phosphatases [53]. On the basis of the analysis of the phospho-proteome dynamics during mitotic exit, this report uncovered a cooperative action of PP2A-Cdc55, PP2A-Rts1, and Cdc14 to ensure the faithful completion of mitosis and suggested a partially overlapping substrate specificity for these three phosphatases [53]. Indeed, despite Cdc14 playing a key role in controlling protein dephosphorylation during mitotic exit, the absence of any of the activities of the aforementioned phosphatases delayed the global amount of these events at the end of mitosis. Following the kinetics of consensus dephosphorylation motifs, this analysis showed that Cdc14 preferentially acts on full pSer Cdk sites, which are dephosphorylated before pThr Cdk motifs. Furthermore, both Cdk sites were shown to be dephosphorylated prior to those motifs recognized and phosphorylated by the polo-like kinase Cdc5 [53]. In contrast to Cdc14, PP2A-Cdc55 preferentially dephosphorylates pThr-Cdk and polo-like motifs, whereas PP2A-Rts1 shows a favored affinity to Aurora kinase substrates [53]. This analysis exemplifies how these proteins are indeed subjected to different levels of regulation that coordinate their activities to ensure, in the case of PP2A and Cdc14, a successful mitotic exit, against the initial misconception that, in contrast to protein kinases, phosphatases are rather promiscuous enzymes in terms of substrate specificity.

## 3. A Role for Cdc14 in the Control of DNA and Centrosome Duplication Cycles and the Maintenance of Genome Stability

Cumulative evidence suggests a conserved function of proteins from the Cdc14 phosphatase family in regulating the centrosome duplication cycle from budding yeast to human cells [12,27,28] (Figure 1). In *S. cerevisiae*, Cdc14 participates in the licensing process of the spindle pole body (SPB), the yeast equivalent of the centrosome, through dephosphorylation of the half-bridge component of this structure Sfi1, and it is further required to avoid SPB re-duplication until completion of mitosis [27,28]. The activity of the phosphatase is necessary for timely SPB duplication, most likely by eliminating inhibitory Cdk1-dependent phosphorylated residues at the C-terminus of Sfi1 [27]. Cdc14-dependent dephosphorylation of Sfi1 mediates its recruitment to initiate a new SPB duplication cycle, this way allowing SPB licensing exclusively during anaphase and thus ensuring that a new SPB replication round will only take place during the next division. Consistent with its role in the regulation of the SPB replication cycle, conditional inactivation of Cdc14 using the temperature-sensitive *cdc14-2* allele delays SPB duplication, whereas overexpression of separase to induce premature activation of the phosphatase drives SPB re-duplication [27,28]. The function of Cdc14 in guiding SPB duplication is also extended to meiosis [87]. Remarkably, in human cells, hCdc14A and hCdc14B seem to have different roles regarding the control of the centrosome duplication cycle. As such, inactivation of hCdc14A in human U2Os cells causes problems during centrosome duplication, whereas overexpression of this isoform leads to premature centrosome disjunction [11]. In contrast, later studies in both HeLa and normal human fibroblasts indicate that hCdc14B depletion causes centriole amplification, whereas increased levels of this Cdc14 paralogue impedes unscheduled centriole duplication in prolonged S phase arrested cells [12].

Cdc14 has also been shown to play an important role during DNA replication (Figure 1). Specifically, this phosphatase seems to display a negative effect on the duplication of the DNA through the dephosphorylation of different replication factors, which prevents new waves of genome doubling taking place until the successful completion of the ongoing replication cycle [43]. Premature release of Cdc14 in cells with compromised Clb5-Cdk1 activity blocks DNA replication due to the early dephosphorylation of Cdk-dependent substrates that include replication factors such as Sld2 and Dpb2 [43]. Another indication of the control exerted by the phosphatase on this process is the observation that, interestingly, Cdc14 also regulates the Swi6 subunit of the SBF (SCB (Swi4/6-dependent cell cycle box) binding factor) and MBF (*Mlu*I binding factor) transcription factors. As such, Swi6 is dephosphorylated in late anaphase to be transported to the nucleus in preparation for the activation during the following G1 phase of SBF- and MBF-dependent genes that participate in membrane and cell wall formation and DNA replication [88,89]. Furthermore, *cdc14-1* mutants display replicative defects that are observed at a genome-wide context but that are specifically relevant at the telomeres and the ribosomal DNA (rDNA) [44]. These defects could be, in fact, originated as a consequence of the downregulation of Swi6-dependent genes, which include the *RFA1* and *RFA2* genes encoding two subunits of the heterotrimeric replication protein A (RPA). Remarkably, Rfa2 is incorrectly localized in the *cdc14-1* mutant [44]. Intriguingly, the DNA replication defects generated in the *cdc14-1* mutant were not detected by the cellular checkpoints, thereby constituting an example of a single point mutation in a regulatory gene with the potential to destabilize the genome and elude the surveillance mechanisms that contribute to maintain DNA integrity. Due to the conservation of the roles of the Cdc14 family of phosphatases, this has important implications for the potential links between deregulation of these proteins and cancer [44].

Besides the regulation of DNA replication, Cdc14 phosphatases also have a direct role in the control of genomic integrity through their participation in the DNA damage response (DDR), which again seems to be conserved across species (Figure 1). As such, after hydroxyurea treatment, the Cdc14 homologue in fission yeast Clp1/Flp1 is translocated from the nucleolus to the nucleus, where it is necessary to promote full activation of the response to replication stress in a process that depends on the DNA damage checkpoint kinase Cds1 [90]. Similarly, mammalian hCdc14A and hCdc14B are necessary for DNA repair, and their depletion leads to the accumulation of γ-H2AX foci and hypersensitivity to ionizing radiation [91,92]. In *S. cerevisiae*, inactivation of Cdc14 causes genomic instability and chromosome rearrangements [93]. Furthermore, Cdc14 release from the nucleolus is also stimulated in budding yeast by exposure to different sources of DNA damage, and its activity is essential to restrain cell cycle progression during repair of the lesions [51]. Interestingly, double strand breaks (DSBs) must be recruited to SPBs to be repaired by homologous recombination, and Cdc14 is also essential for this process, a role that it mediates through dephosphorylation of the SPB component Spc110 [51]. Aside from this role in DSB repair, Cdc14 is also required for the activation of the Yen1 resolvase, which is necessary for the resolution of DNA intermediates that are generated during the repair process [29,30,31]. Although further studies are required to unveil the precise molecular mechanisms by which Cdc14 participates in the DDR, the aforementioned evidence supports the idea that this phosphatase plays a pivotal role in the coordination of the DNA repair process with the successful progression of the cell cycle by intervening in, at least, three key steps of the checkpoint response: first, Cdc14 participates in the activation of the DDR by dephosphorylating the checkpoint kinase Cds1; then, the phosphatase is necessary for DSB repair by promoting anchoring of the lesion to the SPBs through Spc110 dephosphorylation; and finally, by means of the activation of the Yen1 resolvase, it facilitates the resolution of DNA recombination intermediates [92].

## 4. Cdc14 Regulates and is Regulated by rDNA Transcription and Condensation

The activity of Cdc14 is required for the correct segregation of the rDNA, around which the nucleolus is formed [47,94,95,96] (Figure 1). The repetitive nature of the rDNA, which is arranged as a series of tandemly repeated units, makes this genomic region specially challenging in terms of its distribution during mitosis [96,97]. To prevent potential segregation problems, condensation of the rDNA is under a strict control. Intriguingly, rDNA compaction in budding yeast, in contrast to that of the rest of the genome, it is not initiated until the metaphase-to-anaphase transition [47,94,98]. This condensation of the rDNA is promoted by Cdc14 in an Aurora B kinase and condensin-dependent manner [47,94,95,97,99]. Accordingly, several condensin subunits such as Ycs4 [94] or Smc4 [54] are post-translationally modified by Cdc14, and the activity of this phosphatase is required for condensin targeting to the rDNA [48,94]. Indeed, in *cdc14-1* cells arrested at the restrictive temperature, while the rest of the replicated DNA correctly segregates, the rDNA cannot be efficiently distributed between the dividing cells [98]. Again, and interestingly, this function of Cdc14 in rDNA condensation and segregation is also extended to meiosis, where the phosphatase is further required for condensin re-loading at the rDNA during anaphase I [100,101,102]. At the rDNA, Cdc14 also controls rDNA transcription during mitosis. Specifically, once active, Cdc14 inhibits transcription of the rDNA repeats by dephosphorylating RNA polymerase I subunits and thereby precluding its loading on the rDNA [33]. Remarkably, and because transcription interferes with condensin accessibility to the rDNA, this further contributes to the role of Cdc14 in promoting nucleolar condensation [33].

Fascinatingly, we have recently shown that depletion of the small nucleolar ribonucleoprotein particle (snoRNP) assembly factors Hit1 or Rsa1 facilitates increased condensin recruitment to the rDNA by blocking rDNA transcription, and thus induces a premature hyper-condensation of the nucleolus that causes a delay in the FEAR-dependent early release of Cdc14 from this cellular compartment [32]. These results allowed us to propose a simple model that explains how *S. cerevisiae* has elegantly solved the issue of ensuring that the segregation of a structurally problematic genomic region such as the rDNA is coordinated with the precise regulation of mitotic exit through the confinement of Cdc14 in the nucleolus. By placing condensation of the rDNA under the control of the same protein that promotes mitotic exit signaling, cells initiate nucleolar compaction only when the activity of Cdc14 is dynamically promoted and rDNA condensation can occur without interfering with the release of the phosphatase [32]. This further explains why in budding yeast the nucleolus is condensed later than other genomic regions [32]. Excitingly, starvation or rapamycin treatment, which also block rDNA transcription [103,104], lead to similar nucleolar hyper-compaction and defects in Cdc14 release [32]. This fascinating observation opens the door to nucleolar condensation being used a potential mechanism that cells could use to regulate cell cycle progression when facing specific adverse environmental or physiological conditions. Interestingly, like the nucleolus, telomeres also show a delayed segregation with respect to that of the rest of the genome. Remarkably, *cdc14* mutants also display problems with telomere segregation [44,94,105]. Furthermore, Cdc14 activity also promotes condensin loading to these regions by repressing RNA polymerase II transcription through dephosphorylation of its C-terminal domain at sub-telomeric regions, hence facilitating telomere segregation [34]. Notably, and highlighting once again the evolutionary conservation of Cdc14 phosphatases, this function also seems to be maintained in humans [34].

## 5. Cytokinesis is Regulated by Cdc14 Activity and Its Subcellular Localization

As indicated previously, and despite the role of budding yeast Cdc14 in the regulation of mitotic exit being the most thoroughly and extensively studied, the main evolutionary conserved function of this phosphatase probably is, however, the regulation of cytokinesis (Figure 1). In *S. cerevisiae*, Cdc14 localizes at the bud neck after it is released to the cytoplasm in late anaphase. This localization depends on its NES domain and a Crm1/Xpo1-mediated transport [106]. Remarkably, blocking Cdc14 localization to the bud neck compromises actomyosin ring contraction and determines a cytokinesis failure and abnormal re-budding growth [106]. As previously mentioned, the cytokinesis role is more evident for the *S. pombe* Cdc14 homologue, as Clp1/Flp1 is essential for the proper execution of this process but does not play any role in mitotic exit [9,10]. Likewise, silencing Cdc14 homologues in *Caenorhabditis elegans* or hCdc14A in human cell lines also determine cytokinesis problems [11,19,107]. Interestingly, budding yeast Cdc14 dephosphorylates Chs2 (chitin synthase 2) in the endoplasmic reticulum, a step that is required for its delivery to the bud neck, where Chs2 participates in the formation of the primary septum [108], a process that is a basic requirement for actomyosin ring contraction [109]. Furthermore, localization of Cdc14 at the bud neck occurs before splitting of the septin ring and actomyosin ring contraction, and it is important for the dephosphorylation of Inn1, a protein that is required for coupling primary septum formation with actomyosin ring contraction and membrane ingression [110,111]. Besides Inn1, many other Cdc14 substrates that also participate in the coordination of the different stages of the cytokinesis process, such as Aip1, Ede1, Lre1, and Vhs2, have been identified [112,113,114]. Similarly, the guanine exchange factor Bud3, which is necessary to mark the site for bud emergence during the next cell cycle and to direct Clb2 to the bud neck to regulate cytokinesis [115,116,117], is also another Cdc14 phosphatase substrate [54]. One last example of a cytokinesis factor controlled by Cdc14 is Iqg1, whose dephosphorylation by Cdc14 regulates the timing of actin ring assembly and its capacity to be constrained [118].

Interestingly, besides the pool of bud neck-localized Cdc14 substrates with a role in cytokinesis, localization of the phosphatase to the SPBs has been proposed to play an important function during the process of cell division. As such, localization of the Dma1 and Dma2 ubiquitin ligases to the SPB inhibits MEN signaling and prevents septin ring splitting, a cytokinesis block that can be counteracted by Cdc14 loading on the SPBs [85]. Attractively, cytoplasmic release of Cdc14 also seems to facilitate cytokinesis by impeding premature growth polarization towards a new bud site at the end of mitosis [65]. Furthermore, the role of Cdc14 in cytokinesis is also extended to the process of cell separation. The activity of Cdc14 simultaneously counteracts the inhibitory phosphorylation of both the transcription factor Ace2 and the Mob2-Cbk1 kinase, thus promoting the activation of Ace2 by Cbk1 and triggering the Ace2-dependent transcription of genes involved in cell separation [119]. Likewise, the null mutant of Clp1 in *S. pombe* displays endosomal sorting defects, and this phenotype is associated with a negative genetic interaction with mutants in the ESCRT (endosomal sorting complex required for transport) genes, which are required for cell separation [120]. All these observations highlight the pivotal role that Cdc14 plays in the coordination of mitotic exit with the different steps of cytokinesis to ensure a successful separation of the dividing cells.

## 6. Cdc14 Phosphatase and the Defense Against Cellular Stress

Cdc14 not only plays an important role in the DDR, but it is further required during the response to other types of cellular stress. Remarkably, in a global screening for kinase and phosphatases interactors in budding yeast, Cdc14 was shown to display connections with different MAPK (mitogen-activated protein kinases) signaling modules [55]. As such, Cdc14 interacts with the HOG (high-osmolarity glycerol) pathway kinase Pbs2 [55]. In agreement with a connection between Cdc14 and the HOG pathway, overexpression of Cdc14 induces sensitivity to osmotic stress [55], whereas growth under hyper-osmotic conditions promotes a HOG-dependent Cdc14 release from the nucleolus in the absence of MEN pathway signaling [121]. Similarly, Cdc14 also plays an essential nucleating role in the response to sodium chloride (NaCl). Accordingly, Hog1 is hyper-phosphorylated and excluded from the nucleus in *cdc14-3* cells at the restrictive temperature after NaCl treatment [56]. Interestingly, under these conditions, G1 and S phase genes are upregulated, suggesting that the phosphatase is necessary to prevent cell cycle progression under osmotic stress [56]. Indeed, a crucial integrating role has been assigned to this phosphatase connecting the response to osmotic stress mediated by the HOG and CK2 (casein kinase II) signaling pathways with cell cycle regulation [56]. Furthermore, Cdc14 could also bridge regulation of cell cycle progression with the TORC1 (target of rapamycin complex 1) pathway [55,56]. TORC1 signaling plays a fundamental role in coupling nutrient availability with cell metabolism. Remarkably, overexpression of Cdc14 increases the sensitivity of cells to rapamycin, whereas depletion of *CDC14* increases cell resistance to this drug [55]. Inactivation of TORC1 under starvation conditions triggers an autophagy response that is induced by the PP2A-dependent dephosphorylation of Atg13. Loss of PP2A, however, does not completely abrogate Atg13 dephosphorylation in yeast cells [122]. Interestingly, Atg13 contains different phosphorylation motifs, including four pSer-Pro sites that are preferentially targeted by Cdc14 [42,123]. Accordingly, *cdc14-1* cells display a hyper-phosphorylated Atg13 profile, both in normal conditions and under starvation, whereas Cdc14 overexpression or its premature nuclear release by expression of the *net1-1* mutant allele induce autophagy in normal conditions [42]. These observations demonstrate a direct role for the Cdc14 phosphatase in autophagy (Figure 1). Remarkably, this function seems to be also conserved, as suggested by the fact that a *cdc14* null mutant in *Drosophila melanogaster* is defective in its resistance to starvation [124]. As a whole, the cumulative pile of evidence connecting Cdc14 activity with the response to different types of stress strongly suggests that this phosphatase constitutes a central hub for the coordination of cell cycle progression with both intra- and extra-cellular signals that ensures a faithful completion of genome duplication and cell division after the exposure to adverse situations at different cell cycle stages.

## 7. Concluding Remarks

In budding yeast, the ability of the Cdc14 phosphatase to counteract Cdk activity plays an essential role in safeguarding a correct and timely exit from mitosis. However, as highlighted through this review, the additional roles of Cdc14 in the regulation of cell cycle progression and cytokinesis, as well as the coordination of these processes with the faithful duplication of the DNA, the maintenance of the genomic stability, and the even distribution of the chromosomes during mitosis, further make this phosphatase a central regulatory node of a paramount importance for the cells. Cdc14 sequential action impedes new rounds of DNA and SPB duplication until the subsequent cell cycle, promotes cell cycle arrest when the DNA is damaged, facilitates DNA repair by collaborating in the tethering of DSBs to the SPBs, orchestrates rDNA and telomere condensation to ease their segregation during mitosis, and plays a key role in cytokinesis (Figure 1). Additionally, the Cdc14 phosphatase integrates different signaling pathways to coordinate cell cycle progression in response to different types of cellular stresses and environmental cues, such as the cell-wall integrity, the HOG, the pheromone-response, or the filamentous growth signaling pathways [54], and also has the capacity to trigger autophagy under starvation stress [42], which is especially relevant for yeast adaptation to environmental changes once the cell cycle is initiated (Figure 1). Numerous studies demonstrate that many of these functions, if not all, are extraordinarily conserved among the Cdc14 homologues during evolution. Therefore, efforts that aim to uncover new substrates and functions of Cdc14 in *S. cerevisiae* will contribute to shed light in our understanding of the roles that the orthologs of this phosphatase play in human cells.

## Abbreviations

CK2	Casein kinase 2
CDK	Cyclin-dependent kinase
DDR	DNA damage response
DSB	Double strand break
FEAR	Cdc14 early anaphase release pathway
HOG	High-osmolarity glycerol
MAPK	Mitogen-activated protein kinase
MEN	Mitotic exit network
pSer	Phosphorylated serine residue
pThr	Phosphorylated threonine residue
rDNA	Ribosomal DNA
SPB	Spindle pole body
TORC1	Target of rapamycin complex 1
